# Plate reduction in southern Japanese freshwater populations of threespine stickleback (*Gasterosteus aculeatus*)

**DOI:** 10.1002/ece3.10077

**Published:** 2023-05-17

**Authors:** Hiyu Kanbe, Takuya K. Hosoki, Tomoyuki Kokita, Seiichi Mori, Jun Kitano

**Affiliations:** ^1^ Ecological Genetics Laboratory National Institute of Genetics Shizuoka Japan; ^2^ Department of Genetics Sokendai University for Advanced Studies Shizuoka Japan; ^3^ Department of Agriculture Kyushu University Fukuoka Japan; ^4^ Faculty of Economy Gifu‐kyoritsu University Gifu Japan; ^5^ Present address: Field Science Center for Northern Biosphere Hokkaido University Tomakomai Japan

**Keywords:** absorbance, convergent evolution, dissolved organic carbon, iron, non‐parallel

## Abstract

Adaptation to similar environments can lead to the evolution of similar phenotypes in phylogenetically independent lineages. However, the extent of parallel evolution often varies. Because such variations can be due to environmental heterogeneity among seemingly similar habitats, identification of the environmental factors that cause non‐parallel patterns can provide valuable insight into the ecological factors associated with phenotypic diversification. Armor plate reduction in replicate freshwater populations of the threespine stickleback (*Gasterosteus aculeatus*) represents a well‐known example of parallel evolution. Many freshwater populations in multiple regions of the Northern Hemisphere have reduced plate numbers, but not all freshwater populations exhibit plate reduction. In this study, we characterized plate number variation in Japanese freshwater populations and investigated the association between plate number and several abiotic environmental factors. We found that most freshwater populations have not reduced plate numbers in Japan. Plate reduction tends to occur in habitats with warmer winter temperatures at lower latitudes in Japan. In contrast, low dissolved calcium levels or water turbidity had no significant effects on plate reduction, although these were reported to be associated with plate reduction in Europe. Although our data are consistent with the hypothesis that winter temperatures are associated with plate reduction, further studies on the relationship between temperatures and fitness using sticklebacks with varying plate numbers are necessary to confirm this hypothesis and understand the factors causing variations in the extent of parallel evolution.

## Graphical abstract

Reduction of lateral plate number occurs only in southern freshwater populations of threespine stickleback in Japan. Photo by Takuya K. Hosoki.

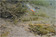

## 1 INTRODUCTION

Adaptation to similar environments can lead to the evolution of similar phenotypes in phylogenetically independent lineages (Schluter, 2000). Such parallel evolution provides evidence for the role of natural selection in phenotypic evolution (Schluter, 2000). However, the extent of parallel evolution often varies, such that the same phenotypes do not always evolve in seemingly similar environments (Bolnick et al., 2018; Fitzpatrick et al., 2014; Heckley et al., 2022; Stuart et al., 2017). Several factors account for variations in the extent of parallel evolution. First, even apparently similar environments may be heterogeneous regarding abiotic and biotic factors associated with a specific phenotype (Fitzpatrick et al., 2014; Stuart et al., 2017). In this case, the varying extent of parallel evolution may provide valuable insight into key environmental factors determining phenotypic variations (Bolnick et al., 2018; Stuart et al., 2017). Second, some populations may not have yet reached an adaptive peak. For example, population sizes may be too small to allow the accumulation of adaptive mutations yet (Crow & Kimura, 1970). Alternatively, the time of exposure to the selective pressure may have been too brief for the population to accumulate adaptive mutations (Crow & Kimura, 1970). Lack of gene flow from a population with adaptive alleles or the presence of gene flow from contrasting environments can also inhibit the population from reaching the adaptive peak (Leinonen et al., 2012; Moore et al., 2007; Stuart et al., 2017). Finally, different populations may use different solutions when exposed to the same selective pressure. There are cases that different phenotypes can exert the same performance and have the same fitness (Bolnick et al., 2018), which is called “many‐to‐one mapping” (Alfaro et al., 2004, 2005; Bolnick et al., 2018; Wainwright et al., 2005).

Armor plate reduction in the threespine stickleback (*Gasterosteus aculeatus*) represents a well‐known example of parallel evolution. While the ancestral marine populations are completely plated with a continuous row of bony plates covering the lateral body surface, many freshwater populations exhibit a reduction in the number of armor plates in multiple regions of the Northern Hemisphere (Bell & Foster, 1994; Wootton, 1984). However, not all freshwater populations have reduced armor plate numbers (Coad & Power, 1974; Edge & Coad, 1983; Hagen & Moodie, 1982; Haines, 2022; Yamasaki et al., 2019). For example, most Japanese freshwater populations reported thus far are completely plated, with only a few low‐plated and partially plated freshwater sticklebacks (Ikeda, 1933; Kitano & Mori, 2016; Yamasaki et al., 2019).

Despite the long history of studies on plate variations in sticklebacks, selective agents of plate reduction remain elusive (Barrett, 2010). Possible abiotic factors favoring plate reduction include low dissolved calcium levels, stained water, low water density, and warm winter temperatures. In freshwater habitats with low dissolved calcium levels, the fish may allocate the rare calcium to growth and other physiological processes essential for survival rather than to plate development (Giles, 1983; Marchinko & Schluter, 2007; Spence et al., 2012). In stained waters, fish can easily hide from predators, which may reduce the adaptive significance of armor plates for postcapture survival (Kitano et al., 2008; Reimchen et al., 2013). In low‐density waters, such as freshwater, where the fish's buoyancy decreases, a reduction in body weight by plate reduction may increase swimming performance and fitness (Myhre & Klepaker, 2009). Although the underlying mechanisms remain unknown, plate reduction tends to occur in habitats with warmer winters (Smith et al., 2020; Wootton, 1976). Biotic factors, such as abundances of different types of predators, may also cause variations in plate reduction (Reimchen, 1994).

In this study, we first conducted sampling of several Japanese freshwater populations whose plate morphs were not reported and characterized plate morph variations in Japan. Next, we collected abiotic environmental data and examined the factors associated with plate variations in the Japanese freshwater populations.

## 2 MATERIALS AND METHODS

### 2.1 Data collection of lateral plate numbers

**FIGURE 1:**
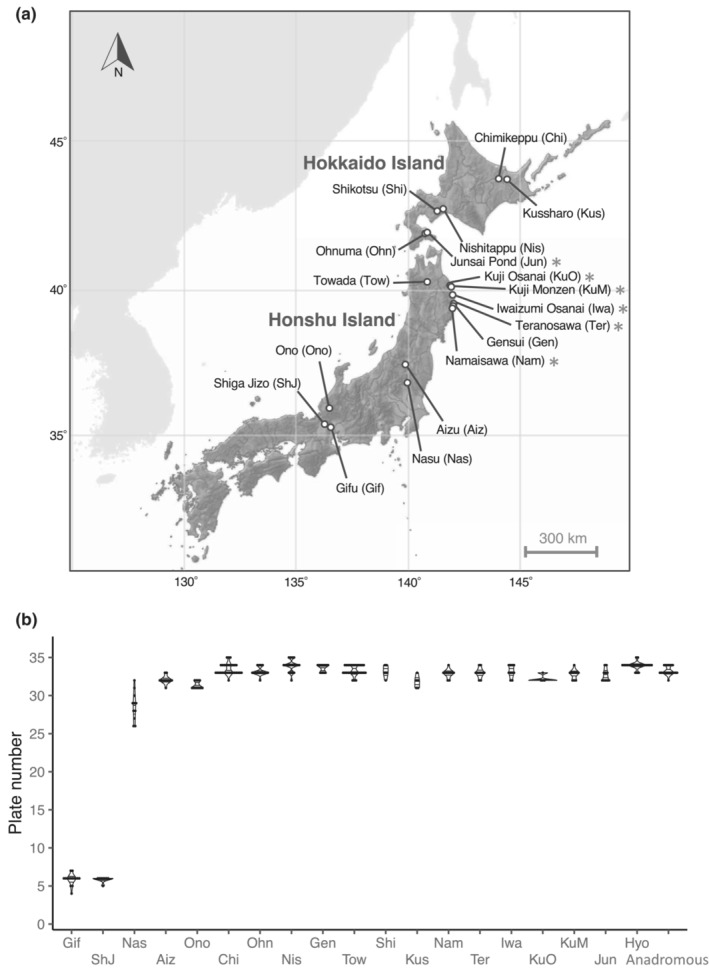
Variation in lateral plate number among Japanese threespine stickleback populations. (a) Sampling sites of freshwater stickleback populations in Japan. The map is based on the Digital Map published by the Geospatial Information Authority of Japan. New habitats analyzed in this study are indicated by asterisks. (b) Violin plot of plate numbers. For the abbreviations of habitats, see (a). Data from one anadromous population from Akkeshi Bay and one brackish‐water resident population from Hyotan Pond published in Kitano et al. (2007) are also plotted for reference.

We collected threespine stickleback from six freshwater habitats (Namaisawa River, Teranosawa River, a stream in Monzen Town in Kuji, Osanai River in Kuji, Osanai River in Iwaizumi, and Junsai Pond; Figure 1a), from which no morphological data had been reported yet (Table S1). The fish were collected using minnow traps. Details of the sampling sites and dates are presented in Table S2. Only fish larger than 32 mm in standard length were included in the analysis, because the lateral plate number reaches its maximum at this body size (Bell et al., 2004). The plate number was counted from the left side of the body under a dissecting microscope. Morphological data for 12 freshwater populations (Gifu, Shiga Jizo, Nasu, Aizu, Ono, Chimikeppu, Ohnuma, Nishitappu, Gensui, Towada, Shikotsu, and Kussharo) were obtained from previous studies (Ishikawa et al., 2019; Kitano et al., 2007; Yamasaki et al., 2019). The population mean was used for analysis because individual‐level data were not available for all populations.

### 2.2 Water measurements

We sampled surface water of stickleback habitats from 2020 to 2021 using a bucket (map in Figure 1a; for details of sampling sites and dates, see Table S1). Before sampling, each bucket was prewashed at least twice with the habitat water. Sampling was not conducted on rainy days. Because the 18 freshwater habitats used for the analysis showed PSU < 0.03, we considered them as freshwater habitats in this study. We collected water samples from several habitats of anadromous populations and brackish‐water habitats, but did not use these data unless noted (Table S1). We measured several parameters (salinity, electrical conductivity, and resistivity) immediately after collection whenever possible. For logistic reasons, including restrictions due to COVID‐19, we could not visit all habitats in person and measure variables at the sampling sites. In such cases, we asked local volunteers to collect water samples and send them to the laboratory. For shipping, the collected water samples were stored in clean polypropylene bottles and sent to the National Institute of Genetics at 4°C. We subsequently measured these parameters using the shipped samples. Next, all water samples were filtered using a 0.7 μm Millipore filter (Whatman) and stored at −30°C until the analysis of other parameters (see below).

For measurement of salinity, electrical conductivity, and resistivity, we used a conductivity meter (HORIBA ES‐51; HORIBA). For the analyses of ions, we measured free calcium ion concentration using the ionic electrometer (LAQUAtwin‐Ca‐11; HORIBA), magnesium ion concentration using the xylidyl blue method (Metallogenics Mg^2+^ measurement LS; Metallogenics Co), and iron ion concentration using a ferrozine assay (Metallogenics Fe^2+^ and Fe^3+^ measurement LS; Metallogenics Co., Ltd). We also measured pH using the pH/ion meter (LAQUA F‐72; HORIBA).

Because plate morph variation may be associated with the axis of stained versus clear waters (Reimchen et al., 2013), we measured water clarity. Absorbance at 340 nm was measured using a microplate reader (FilterMax F5; Molecular Devices). We selected to measure absorbance at 340 nm based on our pilot studies, where we measured absorbance at six wavelengths (250, 340, 405, 450, 595, and 620 nm) of apparently tea‐stained water from a breeding site of anadromous stickleback (Canoe mid site in the Bekanbeushi River: Table S1), which showed the highest absorbance peak at 340 nm (Figure S1). The tea‐stained color of stickleback habitat is generally due to dissolved organic carbon (DOC; Carly et al., 2014; Ormond et al., 2011). To confirm that this is the case with the Japanese habitats, we measured DOC concentrations for several water samples. DOC measurements were conducted by IDEA Consultants using the combustion‐infrared method. To further characterize the substances that make the water stained in the Japanese stickleback habitats, we generated an excitation‐emission map (EEM) at the Horiba Techno Service using a Sievers M9 TOC analyzer (SUEZ Water Technologies & Solutions, Trevose Analytical Laboratory) with the Aqualog (HORIBA). This analysis can reveal detailed profiles of water coloration and fluorescence and, by comparing with those of known substances, we can infer the substances included in the water (Coble, 1996; Senesi et al., 1991).

### 2.3 Temperature and geographical data

Because data on minimum water temperatures were unavailable, we used data on the minimum ambient temperature at the nearest locations, available from the Japan Meteorological Agency database (https://www.data.jma.go.jp/obd/stats/etrn/index.php). We collected the minimum ambient temperature for each year from 2001 to 2021 and calculated the average (Table S1). The latitude of each habitat was obtained using Google Earth Pro (v.7.3.3.7786).

### 2.4 Statistical analysis

For the statistical analysis, we calculated the average of the water measurement data at each site when the measurements were conducted at multiple time points (Table S3). Principal component analysis (PCA) was applied to evaluate the water properties, using the statistical package R version 4.2.2 with standardized data of seven parameters (salinity, conductivity, free calcium ion concentration, magnesium ion concentration, iron ions concentration, absorbance at 340 nm, and pH). These seven parameters were used for PCA because these could be measured for all freshwater water samples. To determine whether the first three PC scores explain the lateral plate number of freshwater sticklebacks, we used a generalized linear model (GLM) with a gamma distribution. We calculated η‐squared values to infer the effect sizes using the R statistical package “effectsize” (Ben‐Shachar et al., 2020).

Using the same water chemistry parameters, we conducted partial least square generalized linear regression (PLSGLR) analysis with a gamma model (Bastien et al., 2005; Bertrand & Maumy, 2023). PLSGLR searches for latent composite variables that can best explain the variance of plate number. We conducted a leave‐one‐out cross‐validation and searched for the number of latent components based on the *Q* ^2^ criterion: a negative *Q* ^2^ value indicates a poor predictive power. We used the R statistical package “plsRglm” (Bertrand & Maumy, 2023).

To determine whether the lateral plate number of freshwater sticklebacks was influenced by free calcium ion concentration, absorbance at 340 nm, winter minimum temperatures and latitude, we used a GLM with a gamma distribution. These parameters were tested because these were reported to be associated with plate numbers in previous studies (see Section 1). To test whether the water variables were correlated with each other, we used Spearman's rank correlation test. We considered *p* < .05 as a significant effect or association in all statistical tests.

## 3 RESULTS

### 3.1 Plate variation among freshwater populations of the threespine stickleback in Japan

All six freshwater populations newly analyzed in this study were completely plated with the mean plate number ranging from 32.25 to 33.2. Combining with previous data, we confirmed that only three freshwater populations (Gifu, Shiga Jizo, and Nasu) were low‐plated or partially plated in Japan (Figure 1b, Table S1).

### 3.2 Habitat water properties of resident sticklebacks in Japan

**FIGURE 2:**
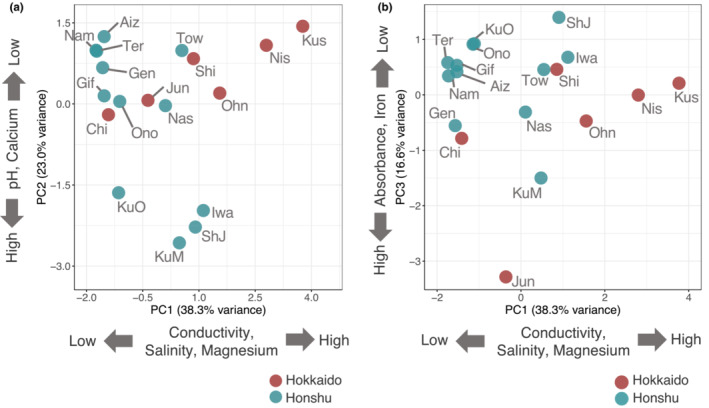
Principal component analysis (PCA) of water measurements of freshwater stickleback habitats in Japan. The left figure is PC1 versus PC2, while the right is PC1 versus PC3. The percentage of variance explained by each PC is shown. Different colors indicate different islands. For the abbreviations of habitats, see Figure 1a.

Using PCA, we visualized variations in the physical and chemical properties of water of freshwater stickleback habitats (Figure 2a). The first (PC1), the second (PC2), and the third (PC3) principal components explained 38.3%, 23.0%, and 16.6% of the variances, respectively (Table S4). Higher PC1 values reflect higher values of salinity, conductivity, free calcium ion concentration, and magnesium ion concentration, suggesting that PC1 reflects the amount of brackish water. The concentrations of free calcium ion and magnesium ion were positively correlated with salinity (Spearman's rank correlation test: *r* = .4793, *p* = .0464 for calcium ion; *r* = .6495, *p* = .0035 for magnesium ion; Figure S2).

Lower PC2 values reflect higher free calcium concentration and higher pH. Although a previous study showed a correlation between dissolved calcium concentration and pH (Smith et al., 2020), the correlation was not significant in our samples (Spearman's rank correlation test: *r* = .0797, *p* = .7532; Figure S2).

Lower PC3 reflects higher absorbance at 340 nm and higher iron ions concentration, suggesting that PC3 reflects the axis of clear versus tea‐stained water. As water turbidity in the North American and European stickleback habitats is caused by DOC (Carly et al., 2014; Ormond et al., 2011), we tested whether water coloration is correlated with the amount of DOC in the Japanese stickleback habitats. We observed a trend for habitats with higher absorbance at 340 nm to have higher DOC values, although the correlation was not statistically significant (Spearman's rank correlation test: *r* = .6236, *p* = .0541; Figure S2). Excitation Emission Matrix analysis of water samples from two stickleback habitats showed a fluorescence peak near the peak of fulvic acids (excitation/emission: 320 nm/430 nm; Nagao et al., 2016); fluvic acids represent a class of humic substances (Peña‐Méndez et al., 2005). This suggests that the stained water is caused by fulvic acids.

### 3.3 Association of lateral plate number with temperature and latitude

**FIGURE 3:**
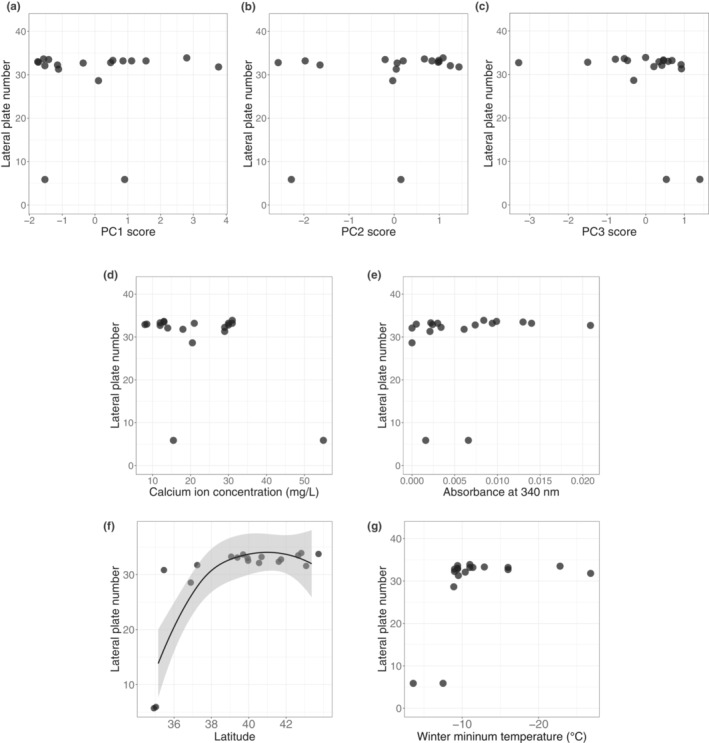
Scatterplots between lateral plate number and several environmental factors. The *Y*‐axis indicates the mean plate number in each freshwater habitat. The *X*‐axis indicates (a) Principal component (PC)1 score, (b) PC2 score, (c) PC3 score, (d) free calcium ion concentration, (e) habitat water absorbance at 340 nm, (f) latitude, and (g) minimum temperature. For visualization, data are fitted with LOESS regression in (f).

There was no significant association between lateral plate number and any PC of the habitat water (GLM: *p* = .8850, coefficient estimate ± SE = 0.02948 ± 0.2002, η^2^ = 0.0023 for PC1; *p* = .8570, coefficient estimate ± SE = 0.05125 ± 0.27877, η^2^ = 0.0431 for PC2; *p* = .7790, coefficient estimate ± SE = −0.08959 ± 0.31300, η^2^ = 0.0426 for PC3; Figure 3a–c). PLSGLR analysis revealed no composite variables with *Q* ^2^ > 0, suggesting that habitat water chemical properties have small effects on the plate number variations.

There was no significant association between lateral plate number and calcium ion concentration (GLM: *p* = .0806, coefficient estimate ± SE = −0.3140 ± 0.1684, η^2^ = 0.0840; Figure 3d). Although we noticed a trend for sticklebacks in the habitats with higher calcium concentrations to have fewer plates, this was contrary to our expectation. There was no significant association between lateral plate number and water absorbance at 340 nm (GLM: *p* = .4590, coefficient estimate ± SE = 286.184 ± 376.724, η^2^ = 0.0143; Figure 3e).

We found a significant association between latitude and number of lateral plates (GLM: *p* = .004, coefficient estimate ± SE = 2.079 ± 0.609, η^2^ = 0.1881; Figure 3f). Reflecting the fact that minimum winter temperature was negatively associated with latitude (GLM: *p* < .001, coefficient estimate ± SE = −0.36509 ± 0.08289; η^2^ = 0.5881; Figure S4), we found a trend of plate number increasing with lower minimum winter temperature (GLM: *p* = .0765, coefficient estimate ± SE = −0.6758 ± 0.3569, η^2^ = 0.0701; Figure 3g).

## 4 DISCUSSION

Our additional sampling of six freshwater populations of the threespine stickleback confirmed that most Japanese freshwater populations are completely plated. Plate reduction tends to occur in habitats with warmer winter temperatures at lower latitudes in Japan, suggesting that temperature may be an important factor influencing the evolution of plate reduction in sticklebacks. Baumgartner and Bell (1984) also reported a similar tendency of southern populations having smaller numbers of plates than northern populations in California within a range of latitude close to that of our study. We also found a trend of sticklebacks in habitats with warmer winter temperatures having smaller numbers of plates, although the trend was not statistically significant. Several previous reports showed a relationship between temperature and plate number (Smith et al., 2020, 2021; Wootton, 1976). Smith et al. (2020) put forward the hypothesis that warmer environments may enhance metabolism and limit body growth, and the armor reduction may be an adaptive strategy for smaller‐sized fish to increase swimming performance (Videler, 1993). Consistent with this hypothesis, stickleback body size is negatively associated with temperature and positively correlated with plate number (Smith et al., 2021). Reimchen (2000) proposed that cold temperatures may reduce escape response and favor armor plates that can increase the post‐capture survival rates. Similar to Japanese populations, most eastern Canadian freshwater stickleback populations are completely plated (Coad & Power, 1974; Edge & Coad, 1983; Hagen & Moodie, 1982; Haines, 2022), which is thought to result from an adaptation to the extremely cold winter conditions (Hagen & Moodie, 1982). However, several low‐plated populations are known to occur in Greenland, a region of extreme cold conditions (Liu et al., 2018). Therefore, how the winter temperature influences the fitness of sticklebacks with varying armor plates remains unknown. Fitness experiments and physiological analyses are necessary to answer these questions.

We found a trend of stickleback populations in habitats with lower calcium concentrations having larger numbers of plate counts. This trend is contrary to what is expected from previous studies. A low dissolved calcium in freshwater habitats is one of the abiotic factors potentially associated with plate reduction in European populations (Giles, 1983; Klepaker et al., 2016; Smith et al., 2020). The dissolved calcium ion concentrations in stickleback habitats in Japan generally appear higher than those of stickleback habitats in Europe (MacColl & Aucott, 2014; Spence et al., 2013). Thus, the known examples of a calcium effect on stickleback plate numbers are in a range of calcium values different from ours. These suggest that selective pressure induced by calcium deficiency is rare in Japanese freshwater habitats. The habitat of the Shiga Jizo population, which is mainly low‐plated, had even the highest dissolved calcium ion concentration (55 mg/L) among the freshwater environments investigated in this study. However, it should be noted that various measurement methods have been used in different studies, and thus, comparisons among different studies need to be discussed with caution (MacColl & Aucott, 2014).

Water clarity, too, was not associated with the plate number. A tendency for plate reduction in turbid water environments has been recorded in Haida Gwaii and North Uist populations (Klepaker et al., 2016; Reimchen et al., 2013). However, DOC in British Columbia (5.0 mg/L on average; 0.7–9.1 mg/L; Ormond et al., 2011) is much higher than that in Japan (0.7 mg/L on average; 0.1–2.9 mg/L), suggesting that selective pressure for plate reduction caused by stained water may be weaker in Japan compared to other geographical regions. Although we did not investigate predator communities in this study, analysis of predators is necessary for knowing the contribution of biotic factors to the plate variation in the Japanese stickleback populations.

Non‐parallel evolution can be caused by factors other than environmental ones. It is possible that several Japanese freshwater populations could not acquire mutations responsible for plate reduction. In North America and Europe, parallel evolution of plate reduction is caused by the repeated fixation of single‐origin freshwater alleles at the *Ectodysplasin* (*Eda*) locus (Colosimo et al., 2005), which is supplied by gene flow (Schluter & Conte, 2009). In Japan, however, no freshwater populations possess the canonical low *Eda* allele (Yamasaki et al., 2019), suggesting that independent de novo mutations are responsible for plate reduction. This may be attributed to the absence of standing genetic variation in the canonical low *Eda* allele in the marine populations around the Japanese archipelago, which has not yet been tested. Furthermore, our previous phylogenomic analysis showed that the Japanese populations with plate reduction colonized freshwater around 100–170 thousand years ago (Kakioka et al., 2020), which predates the colonization of northern Japan by other freshwater populations. Therefore, northern populations may require more time to acquire de novo mutations for reducing armor plates. Further studies on the genetic basis of plate reduction in the Japanese southern freshwater populations and demographic history of freshwater populations will help understand the role of genetic factors in shaping the patterns of parallel evolution.

Here, we characterized plate variation in Japanese freshwater threespine sticklebacks and found that the lower latitudes and warmer winter temperatures may be associated with plate reduction. However, it remains elusive whether this pattern is due to ecological or genetic factors. Further ecological studies on the relationship between environmental factors and fitness using sticklebacks with varying plate numbers are necessary to understand how ecology influences the variation in the extent of parallel evolution.

## AUTHOR CONTRIBUTIONS

**Hiyu Kanbe:** Conceptualization (equal); data curation (lead); formal analysis (lead); investigation (lead); visualization (lead); writing – original draft (lead); writing – review and editing (supporting). **Takuya K. Hosoki:** Data curation (supporting); resources (supporting); writing – review and editing (supporting). **Tomoyuki Kokita:** Data curation (supporting); resources (supporting); writing – review and editing (supporting). **Seiichi Mori:** Data curation (supporting); resources (supporting); writing – review and editing (supporting). **Jun Kitano:** Conceptualization (equal); funding acquisition (lead); investigation (supporting); supervision (lead); writing – original draft (supporting); writing – review and editing (lead).

## CONFLICT OF INTEREST STATEMENT

The authors have no competing interests to declare.

## Supporting information


Data S1.

Data S2.

## Data Availability

All data used for the analysis in this paper are included in the supplementary tables.
