# Correction to “Plate Reduction in Southern Japanese Freshwater Populations of Threespine Stickleback (*Gasterosteus aculeatus*)”

**DOI:** 10.1002/ece3.71052

**Published:** 2025-03-12

Kanbe, H., Hosoki, T. K., Kokita, T., Mori, S., and Kitano, J. (2023). Plate reduction in southern Japanese freshwater populations of threespine stickleback ( *Gasterosteus aculeatus* ). *Ecology and Evolution*, 13, e10077. https://doi.org/10.1002/ece3.10077

We have shown all salinity data as %, but we have mistakenly referred to it as PSU. The number should be multiplied by 10 to get PSU. Corrected versions of Figure S2, Table S2, and Table S3 have been updated in the Supporting Information of the original article.

The text in “2.2 Water measurements” of the Materials and Methods section should be corrected to read as follows: “Because the 18 freshwater habitats used for the analysis showed PSU < 0.3, we considered them as freshwater habitats in this study”. These corrections do not alter the results and conclusions of the paper.

We apologize for this error.

