# Supplementary figures and images for: Plate reduction in southern Japanese freshwater populations of threespine stickleback (Gasterosteus aculeatus)
Source: Ecol Evol. 2023 May 17;13(5):e10077. doi: 10.1002/ece3.10077 (PMC10191778; doi:10.1002/ece3.10077)

Figure.S1

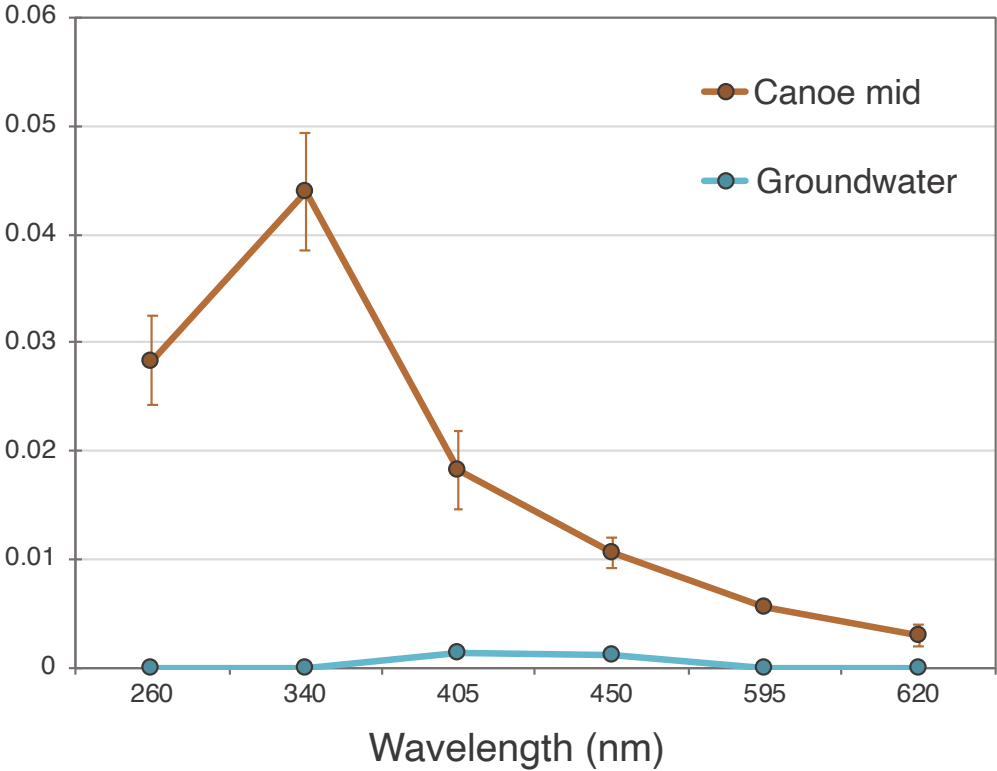

Figure.S2

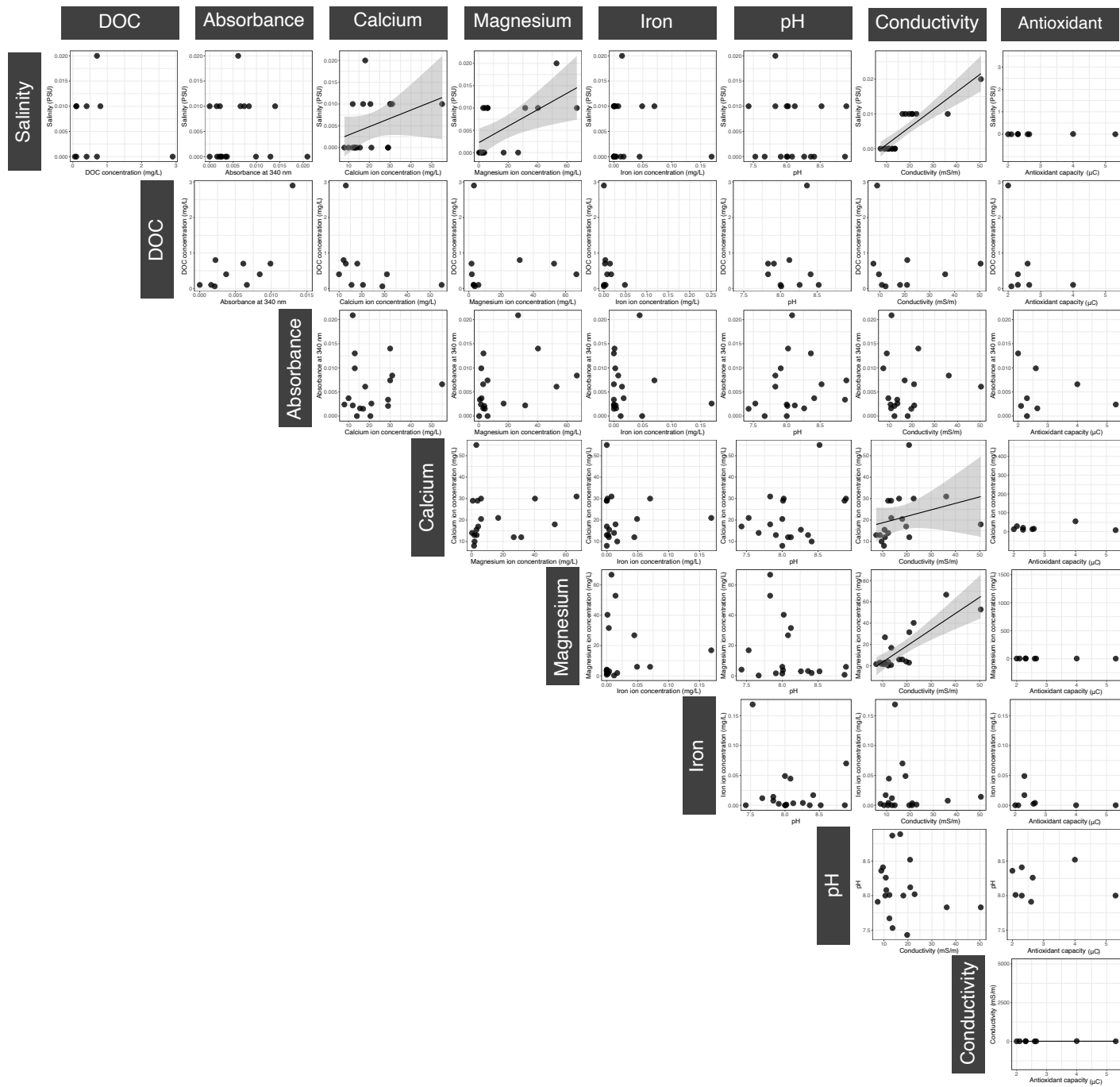

Figure.S3

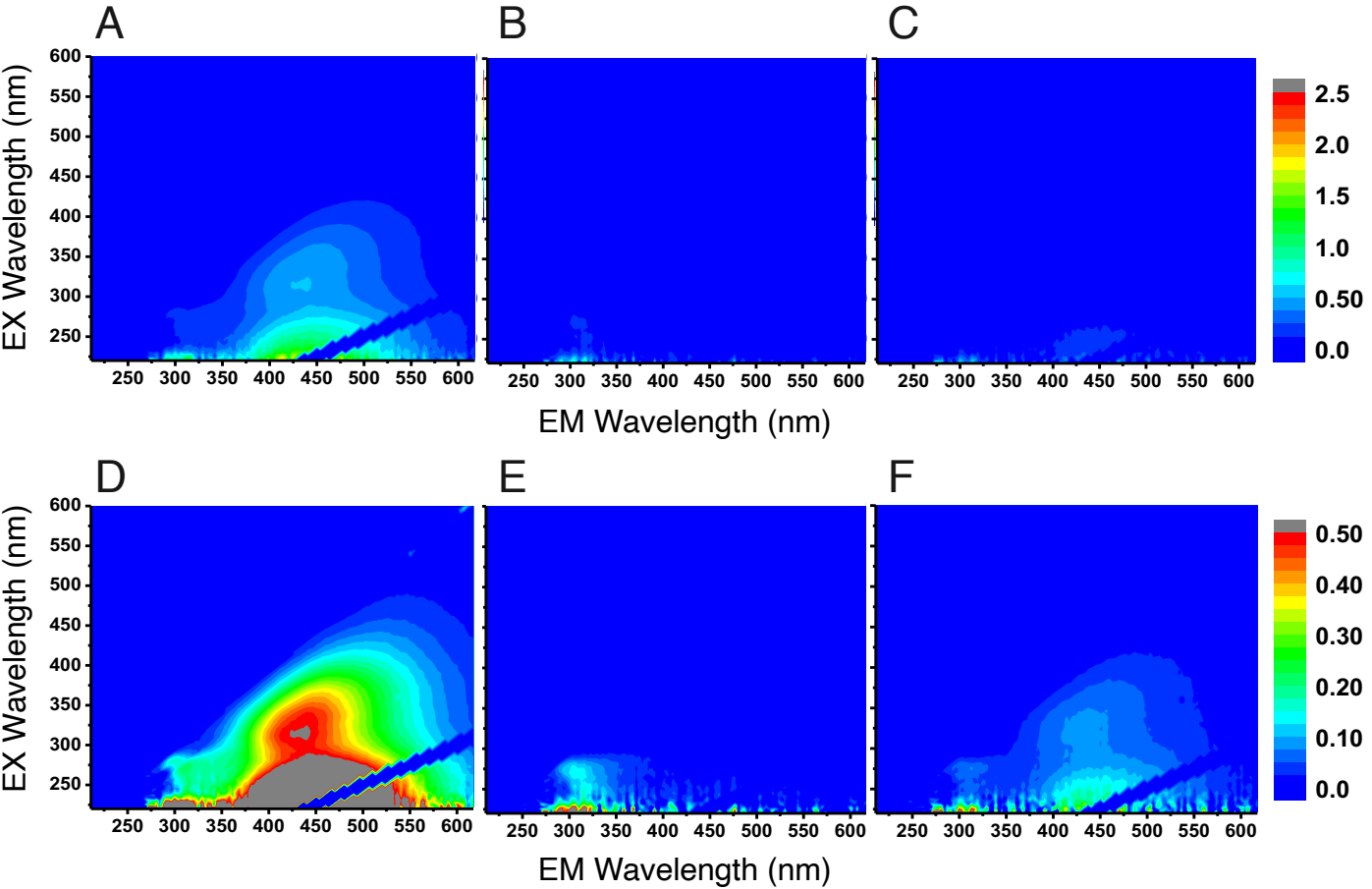

Figure.S4

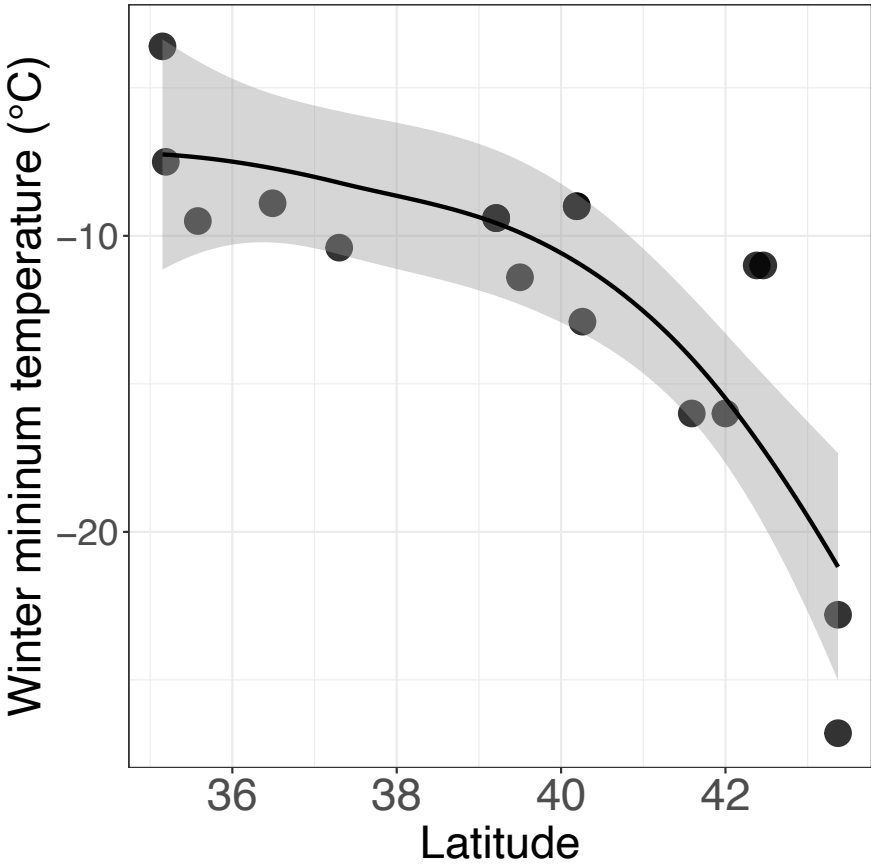

Supplement: Supplementary file 1 — Data S1. [file ECE3-13-e10077-s002.pdf]
